# Sinomenine hydrochloride injection for knee osteoarthritis: A protocol for systematic review and meta-analysis

**DOI:** 10.1097/MD.0000000000028503

**Published:** 2022-01-14

**Authors:** Zeling Huang, Xiao Mao, Junming Chen, Junjun He, Shanni Shi, Miao Gui, Hongjian Gao, Zhenqiang Hong

**Affiliations:** a Fujian University of Traditional Chinese Medicine, Fuzhou, Fujian, China; b Zhoupu Community Health Service Center of XiHu District of Hangzhou, Hangzhou, Zhejiang, China; c Key Laboratory of Orthopedics & Traumatology of Traditional Chinese Medicine and Rehabilitation Ministry of Education (Fujian University of TCM), Fuzhou, Fujian, China.

**Keywords:** knee osteoarthritis, protocol, sinomenine, systematic review, traditional chinese medicine ;

## Abstract

**Background:**

Knee osteoarthritis (KOA) is a degenerative disease in the knee joint, with chronic joint pain, swelling, stiffness, and dysfunction as the primary manifestations. Sinomenine hydrochloride injection is a proprietary Chinese medicine injection of sinomenine, the main active component of traditional Chinese medicine (TCM). Clinical studies show that Sinomenine hydrochloride injection has a good effect on the treatment of KOA. At present, there is still a lack of systematic reviews and meta-analyses to evaluate the efficacy and safety of sinomenine hydrochloride injection in the treatment of KOA. Our purpose is to supplement this deficiency.

**Methods:**

Randomized controlled trials of sinomenine hydrochloride injection in the treatment of KOA were searched for Eight electronic resource databases. We will use Review Manager 5.3 software for heterogeneity assessment, meta-analysis, and subgroup analysis. We will use the Cochrane Manual to assess the quality of the included studies, and use reporting biases assessment and sensitivity analysis to evaluate the reliability and stability of the results.

**Results:**

This study will provide a high-quality synthesis to assess the efficacy and safety of sinomenine hydrochloride injection in the treatment of KOA.

**Conclusion:**

This systematic review evaluates the efficacy and safety of sinomenine hydrochloride injection in the treatment of KOA.

**INPLASY registration number:**

INPLASY2021110057.

## 1 Introduction

Osteoarthritis (OA) is one of the primary diseases affecting human health. It involves multiple joints and commonly affects the knee and hip joints. The pathological changes of Knee Osteoarthritis (KOA) are mainly the gradual degeneration of hyaloid cartilage and surrounding tissues (including ligaments, synovium, and subchondral bone) of the knee, resulting in joint pain, enlargement, stiffness and dysfunction, and even disability in severe cases.^[1]^ With the increase in life expectancy and aging of the global population, its incidence is increasing, and the burden on countries worldwide is consequently becoming greater.^[2]^ At present, the drugs used to treat KOA mainly include analgesics, intraarticular corticosteroids, non-steroidal anti-inflammatory drugs (NSAIDs), and symptomatic slow-acting drugs for osteoarthritis. Although these drugs have specific effects for OA patients, they also increase the incidence of gastrointestinal ulcers and cardiovascular events, affecting their use by some patients.^[3]^

Traditional Chinese medicine (TCM) has a long history and has the advantages of natural curative effect, safety, and stability. It is a research hotspot in the treatment of many complex diseases.^[4]^ Sinomenine (chemical structure: C19H23NO4) is a monomer alkaloid extracted from the TCM Sinomeniumacutum, with anti-inflammatory, analgesic, and immunomodulatory effects.^[5–8]^ Sinomenine hydrochloride injection is a sterilized aqueous solution made of sinomenine, which can be directly injected into the affected area through the joint cavity and exert the pharmacological effect of sinomenine. At present, there are many clinical research reports on the treatment of KOA by intra-articular injection of sinomenine hydrochloride.^[9–14]^ However, no systematic evaluation has been published on this issue, and it is not clear whether sinomenine hydrochloride injection is effective and safe in treating KOA. Therefore, it is essential to conduct a systematic evaluation to obtain relatively convincing conclusions as to whether sinomenine hydrochloride injection can be a good choice as a complementary and alternative drug (CAM) for KOA.

## 2 Methods

This protocol is registered with INPLASY(INPLASY2021110057; DOI number10.37766/inplasy 2021.11.0057). Because this study only reorganizes and analyzes all data related to clinical trials, ethical approval is unnecessary.

### 2.1 Inclusion and exclusion criteria for study selection

#### 2.1.1 Types of studies

The RCTs are eligible, whether or not the blind method is specifically described. There are no restrictions on languages. In addition, we will exclude systematic reviews, review literature, and literature whose full text is not available.

#### 2.1.2 Types of participants

Patients should be diagnosed with KOA. No restrictions on country, race, age, or gender.

#### 2.1.3 Type of interventions

##### 2.1.3.1 Control interventions

Sodium hyaluronate, triamcinolone acetonide, and other drugs were injected into the joint cavity.

##### 2.1.3.2 Experimental interventions

Based on the control group, sinomenine hydrochloride injection was combined with joint cavity injection, or used sinomenine hydrochloride injection alone.

#### 2.1.4 Types of outcome measures

##### 2.1.4.1 Primary outcomes

Total effective rate, Visual Analog Scale (VAS), Western Ontario and Mc Master University (WOMAC) Osteoarthritis Index.

##### 2.1.4.2 Secondary outcomes

Circumference of the knee joint, the knee joint fluid or serum interleukin-1β (IL-1β) level, the knee joint fluid or serum tumor necrosis factor-α (TNF-α) level, adverse events.

### 2.2 Search methods for identification of studies

Randomized controlled trials (RCTs) of sinomenine hydrochloride injection in the treatment of KOA were searched in PubMed, Web of Science, Embase, Cochrane Library, Allied and Complementary Medicine Database (AMED), China National Knowledge Infrastructure (CNKI), China Science and Technology Journal Database (VIP), Wanfang Database. The retrieval time is from a database construction to the present. Different search methods will be adjusted according to different Chinese and English databases. The search strategy was as follows, taking PubMed as an example (Table 1).

**Table 1:** Search strategy for PubMed.

Number	Terms
#1	knee osteoarthritis [ti, ab]
#2	osteoarthritis of knee [ti, ab]
#3	knee pain [ti, ab]
#4	knee joint osteoarthritis [ti, ab]
#5	knee arthritis [ti, ab]
#6	KOA [ti, ab]
#7	#1 or #2–6
#8	sinomenine hydrochloride injection [ti, ab]
#9	Zhengqing Fengtongning injection [ti, ab]
#10	Zhengqing Fengtongning [ti, ab]
#11	Sinomenine [ti, ab]
#12	#8 or #9–11
#13	the knee joint cavity [ti, ab]
#14	knee joint [ti, ab]
#15	Joint cavity injection [ti, ab]
#16	#13 or #14–15
#17	#7 and #12 and #16

### 2.3 Data selection

**Figure 1:**
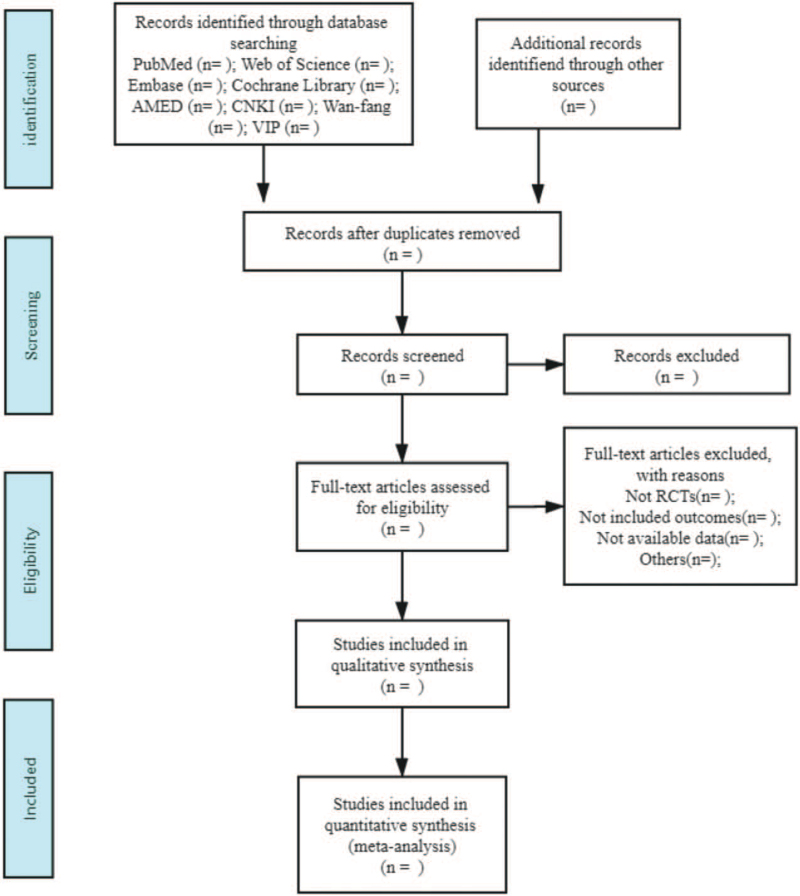
Flow diagram of the study selection process.

The two researchers will import all the retrieved articles into Endnote X9, and use the software to filter and delete duplicate data. Then according to the inclusion and exclusion criteria, the literature was screened out, and the data was extracted independently. The extraction includes information such as the first author, publication year, baseline characteristics of the research object, and outcome indicators, and cross-check the extracted data after completion. In a disagreement, the two researchers discuss it or make a joint decision by a third-party researcher with a senior professional title (Fig. 1).

### 2.4 Quality Assessment of the Included Studies

Bias risk assessment was conducted by two reviewers based on the bias risk assessment tool recommended in the Cochrane Manual.^[15]^

### 2.5 Statistical Analysis

Review Manager (Revman), version 5.3, was used to analyze the collected clinical research data. According to the difference of enumeration data and measurement data, the distribution was evaluated by relative risk (RR) and standardized mean difference (SMD), and the confidence interval (95% confidence interval, CI) was 95%. If the heterogeneity I^2^ < 50%, the heterogeneity among the included studies was considered minor, and the fixed effect model was adopted. If I^2^ ≥ 50%, the heterogeneity among the included studies was deemed significant, and the random effect model was adopted.^[16]^ Subgroup analysis was conducted according to different treatments in the treatment group, and sensitivity analysis was also used to analyze the sources of heterogeneity. A value *P* < .10 was considered to suggest statistical heterogeneity.

### 2.6 Subgroup analysis

We will use subgroup analysis based on different interventions and controls. This can analyze the sources of heterogeneity and enhance the persuasiveness of the conclusions.

### 2.7 Assessment of reporting biases

Funnel plot^[17]^ and Egger regression test^[18]^ were used to determine potential reporting bias.

### 2.8 Sensitivity analysis

We will conduct sensitivity analysis by removing one study one by one, and investigate the reliability and stability of the results.

## 3 Discussion

*Sinomeniumacutum* is a TCM, which has the function of dispelling wind dampness, channeling channels, and collaterals, relieving urination. *Sinomeniumacutum* is often used to treat rheumatoid arthritis, OA, and gout arthritis.^[19]^ Sinomenine is the main active ingredient of *Sinomeniumacutum*. Studies have found that KOA is a chronic inflammatory process involving inflammatory mediators and a variety of cytokines. Cytokines TNF-α and IL-1β can inhibit cartilage matrix synthesis and promote cartilage degeneration and decline. Articular cartilage degeneration is the earliest and most important structural change of OA, including significant chondrocyte apoptosis and excessive extracellular matrix (ECM) degradation. The apoptosis of many chondrocytes reduces the number of cells in the cartilage tissue, weakens the ability to synthesize matrix, and leads to the synthesis and release of matrix-degrading enzymes, which further damages the cartilage matrix.^[1]^ Therefore, many drugs play a role in delaying the progression of OA by inhibiting the expression of inflammatory factors and chondrocyte apoptosis. Studies have shown that sinomenine can reduce synovial inflammation and cartilage degeneration by inhibiting the expression of inflammatory factors and chondrocyte apoptosis, thus delaying the progression of osteoarthritis.^[20]^ Sinomenine hydrochloride injection as an external preparation of sinomenine has been used ever more frequently in the clinical treatment of OA. This study gives an insight on whether sinomenine injection is efficacious and safe in the treatment of KOA, which will be useful to KOA patients, physicians, and decision-makers.

## Author contributions

**Conceptualization:** Zeling Huang, Xiao Mao, Zhenqiang Hong.

**Data curation:** Zeling Huang, Xiao Mao, Junming Chen.

**Formal analysis:** Junjun He, Shanni Shi, Miao Gui.

**Investigation:** Zeling Huang, Xiao Mao.

**Supervision:** Hongjian Gao.

**Writing – original draft:** Zeling Huang, Xiao Mao.

**Writing – review & editing:** Zhenqiang Hong.
